# Calmodulin Regulates Transient Receptor Potential TRPM3 and TRPM8-Induced Gene Transcription

**DOI:** 10.3390/ijms24097902

**Published:** 2023-04-26

**Authors:** Gerald Thiel, Oliver G. Rössler

**Affiliations:** Department of Medical Biochemistry and Molecular Biology, Saarland University, Building 44, 66421 Homburg, Germany

**Keywords:** calmodulin, calcineurin, ophiobolin A, TRPM3, TRPM8

## Abstract

Calmodulin is a small protein that binds Ca^2+^ ions via four EF-hand motifs. The Ca^2+^/calmodulin complex as well as Ca^2+^-free calmodulin regulate the activities of numerous enzymes and ion channels. Here, we used genetic and pharmacological tools to study the functional role of calmodulin in regulating signal transduction of TRPM3 and TRPM8 channels. Both TRPM3 and TRPM8 are important regulators of thermosensation. Gene transcription triggered by stimulation of TRPM3 or TRPM8 channels was significantly impaired in cells expressing a calmodulin mutant with mutations in all four EF-hand Ca^2+^ binding motifs. Similarly, incubation of cells with the calmodulin inhibitor ophiobolin A reduced TRPM3 and TRPM8-induced signaling. The Ca^2+^/calmodulin-dependent protein phosphatase calcineurin was shown to negatively regulate TRPM3-induced gene transcription. Here, we show that TRPM8-induced transcription is also regulated by calcineurin. We propose that calmodulin plays a dual role in regulating TRPM3 and TRPM8 functions. Calmodulin is required for the activation of TRPM3 and TRPM8-induced intracellular signaling, most likely through a direct interaction with the channels. Ca^2+^ influx through TRPM3 and TRPM8 feeds back to TRPM3 and TRPM8-induced signaling by activation of the calmodulin-regulated enzyme calcineurin, which acts as a negative feedback loop for both TRPM3 and TRPM8 channel signaling.

## 1. Introduction

Calmodulin is a small, acidic protein that functions as a Ca^2+^ binding molecule with four EF-hand motifs. Calmodulin controls the activity of numerous enzymes, including phosphorylase kinase, calcineurin, Ca^2+^/calmodulin-dependent protein kinases, and endothelium-derived nitric oxide synthase. Calmodulin is involved in the regulation of ion channels, as reported for Ca^2+^-activated potassium channels [1,2], *ether à go-go* K^+^ channels [3], KCNQ M-type K^+^ channels [4,5], and calcium-activated Cl^−^ channels [6]. A calmodulin-binding “IQ” motif has been discovered in the C-terminal domain of the α1 subunit of voltage-gated L-type Ca^2+^ channels. Calmodulin functions as a Ca^2+^ sensor and regulates both Ca^2+^-dependent inactivation and facilitation of voltage-gated Ca^2+^ channels [7,8]. The binding of Ca^2+^/calmodulin to voltage-gated Ca^2+^ channels is also required for the initiation of a signaling cascade leading to the activation of gene transcription following stimulation [9].

Calmodulin has been reported to regulate positively or negatively the biological activities of numerous transient receptor potential (TRP) channels [10], proteins involved in the regulation of thermosensitivity, pain sensation, perception of pungent chemicals, secretion, cell proliferation, tumorigenesis, and neurotransmission [11]. TRPC6 channels bind calmodulin, resulting in the suppression of TRPC6 activity. This TRPC6/calmodulin interaction is disrupted by phosphoinositides [12]. Other studies have reported that inhibition of calmodulin function attenuates TRPC6 channel activation [13,14], i.e., calmodulin would be required for TRPC6 channel activation in this case. Calmodulin interacts with an “IQ”-like motif in the N-terminal region of the TRPM2 channel and supports channel activation [15]. Within the TRPV1 channel, calmodulin binds to a cytosolic ankyrin repeat domain and competes with ATP and phosphatidylinositol 4,5-bisphosphate for binding. Function experiments have shown that calmodulin is required for the desensitization of the channel after repeated stimulation (tachyphylaxis), whereas ATP and phosphatidylinositol 4,5-bisphosphate exert opposite activity [16]. Ca^2+^/calmodulin binds directly to an intracellular domain of the TRPV4 channel, which is a prerequisite for TRPV4 activation [17]. Calmodulin also binds to TRPV6 channels and positively regulates TRPV6 activity [18]. Another study proposed that overexpression of calmodulin reduced TRPV6-derived currents [19].

In this study, we investigated by genetic and pharmacological means the functional role of calmodulin in modulating intracellular signaling and gene transcription triggered by activation of TRPM3 or TRPM8 channels. It has been proposed that the biological activities of both TRP channels are regulated by calmodulin [20,21,22]. TRPM3 channel activation has been associated with temperature and pain sensation, secretion, muscle contraction, tumorigenesis, and gene transcription [23]. TRPM3, together with TRPA1 and TRPV1 channels, regulates heat sensation in the somatosensory system [24].

TRPM8 was originally described as a menthol receptor because menthol and other cooling substances such as eucalyptol and the synthetic super-cooling agent icilin induce Ca^2+^ influx through this channel. TRPM8 detects cold from the environment and acts as a nociceptor for cold-sensing dorsal root, trigeminal, and ganglion neurons. It has been described to play an important role in regulating cold-induced pain [25,26,27].

Given the important physiological role of TRPM3 and TRPM8 channels, it is of particular scientific interest to understand how channel activation induces biochemical and physiological changes in cells. To this end, the signaling molecules that link channel activation to physiological functions need to be identified. Because TRP channels can function as Ca^2+^ channels, it is obvious to investigate the role of the Ca^2+^ binding protein calmodulin in regulating TRPM3 and TRPM8 channel functions. Several binding sites for calmodulin have been identified within the TRPM3 channel in vitro [21,22]. Mutation of a particular calmodulin binding site of TRPM3 has been shown to reduce TRPM3 stability [22]. A calmodulin binding site in the *N*-terminal domain of TRPM8 was mapped using in vitro pull-down experiments [28]. Pharmacological inhibition of calmodulin led to conflicting results [20,28,29]. Thus, TRPM3 and TRPM8 signaling may depend on calmodulin, but functional studies are lacking. Therefore, we investigated the functional role of calmodulin in regulating signal transduction and gene transcription of TRPM3 and TRPM8 channels. Our results show that inhibition of calmodulin, either by expression of a calmodulin mutant or by application of a calmodulin inhibitor, reduced TRPM3 and TRPM8 signaling that leads to the activation of the transcription factor AP-1. Calmodulin is also required for the activation of the Ca^2+^/calmodulin-dependent protein phosphatase calcineurin, which acts as a negative regulator of TRPM3 and TRPM8 signaling. Thus, calmodulin plays in this scenario a double role: it is required for TRPM3 and TRPM8 signaling and TRPM3 and TRPM8-mediated gene transcription. Calmodulin also activates calcineurin, which functions as a negative feedback loop of TRPM3 and TRPM8 signaling.

## 2. Results

### 2.1. Expression of a Ca^2+^-Insensitive Calmodulin Mutant Reduces Gene Transcription Induced by Stimulation of TRPM3 Channels with Pregnenolone Sulfate

**Figure 1:**
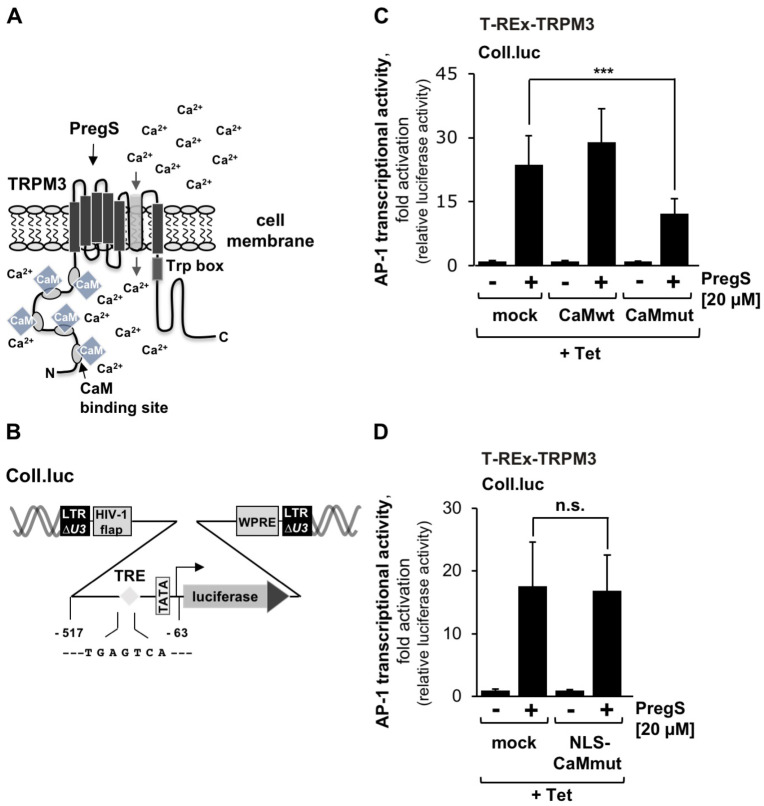
Expression of a calmodulin mutant that does not bind Ca^2+^ ions reduces TRPM3-induced gene transcription. (**A**) Modular structure of TRPM3 showing several calmodulin-binding domains within the *N*-terminal domain. (**B**) Provirus with the Coll.luc reporter gene used as a sensor to measure AP-1 activity. AP-1 binds to the TRE genetic element with the sequence 5′–TGAGTCA–3′. (**C**) T-REx-TRPM3 cells were infected with a lentivirus containing the Coll.luc reporter gene. Cells were additionally infected with a lentivirus encoding either wild-type calmodulin (CaMwt), a calmodulin mutant (CaMmut), or β-galactosidase (mock) as a control. Cells were serum-starved for 24 h in the presence of tetracycline (1 μg/mL) to induce TRPM3 expression. Serum-starved cells were stimulated with pregnenolone sulfate (20 μM) for 24 h. Cell extracts were prepared. Luciferase activities and protein concentrations were determined. Luciferase activities were normalized to protein concentrations. Data presented are means ± SD of three experiments performed in quadruplicate (*** *p* < 0.001; n.s., not significant). (**D**) T-REx-TRPM3 cells containing a chromatin-integrated Coll.luc reporter gene were infected with a lentivirus encoding an NLS containing calmodulin mutant (NLS-CaMmut), or β-galactosidase (mock) as a control. Cells were serum-starved for 24 h in the presence of tetracycline (1 μg/mL) to induce TRPM3 expression. Serum-starved cells were stimulated with pregnenolone sulfate (20 μM) for 24 h. (*n* = 4; n.s., not significant).

The aim of this study was to investigate whether calmodulin is required for TRPM3 signaling. Figure 1A shows the molecular structure of the TRPM3 channel, which has the typical TRP family structure with six transmembrane domains, an ion pore between the fifth and sixth transmembrane domains, and *N*-terminal and *C*-terminal domains located in the cytosol. Five calmodulin binding sites have been mapped in vitro in the extended *N*-terminal domain of TRPM3 [22], but the functional consequences of calmodulin binding are currently unknown. TRPM3 signaling can be measured by analyzing intracellular Ca^2+^ levels, phosphorylation of extracellular signal-regulated protein kinase (ERK1/2), or transcriptional activation. We chose to measure transcriptional activation of the transcription factor AP-1 after TRPM3 stimulation. In this case, signal transduction from the plasma membrane to the nucleus has occurred. As a sensor for AP-1 activation, we used a collagenase promoter/luciferase reporter gene (Coll.luc, Figure 1B), which was integrated into the chromatin of the cells via lentiviral gene transfer [30]. This strategy ensured that the reporter gene was integrated into a nucleosomal structure. T-REx-TRPM3 cells, modified HEK293 cells, were used as a cellular model system because TRPM3 expression could be induced in these cells by adding tetracycline to the culture medium [31]. T-REx-TRPM3 cells were infected with a lentivirus containing the Coll.luc reporter gene. In addition, we infected the cells with a lentivirus encoding wild-type (CaMwt) or mutated (CaMmut) calmodulin. The mutated calmodulin contained point mutations in all four Ca^2+^ binding sites, making it Ca^2+^-insensitive. The mutant competes with wild-type calmodulin for binding to calmodulin targets and therefore functions as a dominant-negative. This approach has also been taken by other investigators analyzing the regulatory role of calmodulin for numerous ion channels [1,2,4,5,6,7,8,9,13,14,18,32,33,34,35]. As a control, cells were infected with a lentivirus encoding β-galactosidase (mock). TRPM3 channels were stimulated with the steroid pregnenolone sulfate [31]. Figure 1C shows that expression of the Ca^2+^-insensitive calmodulin mutant significantly reduced pregnenolone sulfate-mediated activation of AP-1. AP-1 activity was reduced by 48.1%. In contrast, the expression of wild-type calmodulin had no effect on TRPM3-induced signaling and gene transcription. These results indicate that calmodulin is required for TRPM3 channel-induced signal transduction.

### 2.2. Expression of a Ca^2+^-Insensitive Calmodulin Mutant in the Nucleus Has No Effect on TRPM3-Induced Gene Transcription

Several reports have described the translocation of calmodulin into the nucleus following an increase in cytoplasmic Ca^2+^ [36,37]. In the nucleus, calmodulin activates the Ca^2+^/calmodulin-dependent protein kinase CaMKIV and in this way regulates gene transcription mediated by the transcription factor CREB. We, therefore, examined whether expression of the Ca^2+^-insensitive calmodulin mutant in the nucleus affects TRPM3-induced activation of AP-1. Figure 1D shows that expression of this mutant did not reduce pregnenolone-sulfate-mediated stimulation of AP-1, suggesting that calmodulin binds to nonnuclear targets to regulate TRPM3 signaling.

### 2.3. Calmodulin Targets Are Upstream of Raf within the TRPM3-Induced Signaling Cascade

**Figure 2:**
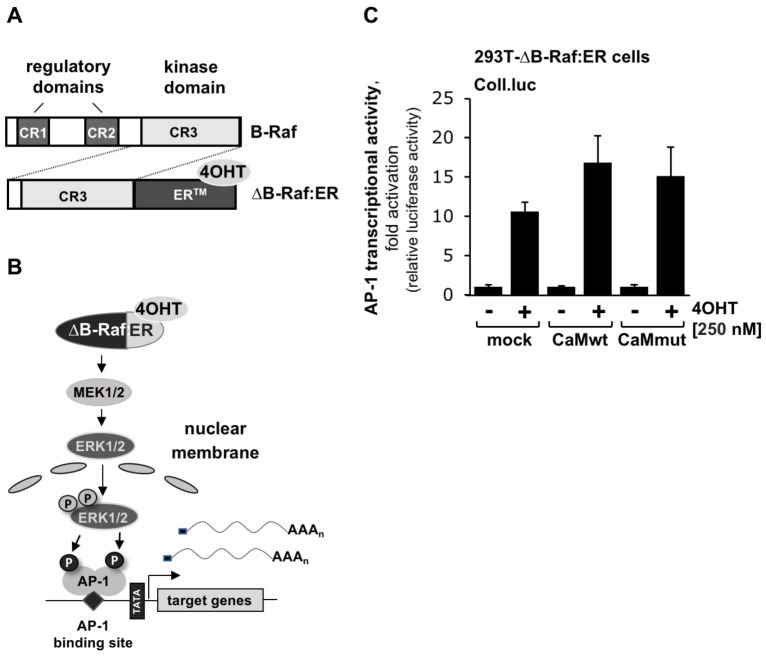
Expression of wild-type calmodulin or Ca^2+^-insensitive calmodulin does not alter B-Raf-induced activation of AP-1 (**A**) Domain structure of B–Raf and the B–Raf mutant ΔB–Raf:ER. (**B**) Signaling pathway linking B–Raf to AP-1. (**C**) HEK293-ΔB–Raf:ER cells containing the Coll.luc reporter gene were infected with a recombinant lentivirus encoding either Ca^2+^-sensitive (CaMwt) or Ca^2+^-insensitive calmodulin (CaMmut). As a control, cells were infected with a lentivirus encoding β-galactosidase (mock). The cells were maintained in a medium containing 0.05% serum for 24 h and then stimulated with 4OHT (100 nM) for 24 h. Cells were harvested and analyzed as described in the legend to Figure 1 (*n* = 3).

Activation of gene transcription after stimulation of TRPM3 channels involves the activation of the ERK1/2 pathway [38]. Similarly, stimulation of L-type voltage-gated Ca^2+^ channels is associated with the activation of ERK1/2. It has been shown that phosphorylation and activation of ERK1/2 is significantly impaired in the presence of a Ca^2+^-insensitive calmodulin mutant [9]. We tested whether the expression of this mutant has a direct effect on the ERK1/2 signaling pathway. We expressed a constitutively active B-Raf protein kinase mutant consisting of the catalytic domain of B-Raf and the ligand binding domain of the estrogen receptor (Figure 2A). This fusion protein can be activated with the compound 4-hydroxytamoxifen (4OHT), resulting in the phosphorylation of MEK1/2, which in turn phosphorylates and activates ERK1/2. The activated ERK1/2 migrates into the nucleus and regulates gene transcription via the phosphorylation of gene regulatory proteins (Figure 2B). Figure 2C shows that the expression of wild-type calmodulin (CaMwt) or its Ca^2+^-insensitive mutant (CaMmut) had no effect on the B-Raf-induced activation of AP-1. These data indicate that calmodulin targets proteins upstream of B-Raf within the TRPM3-initiated signaling cascade.

### 2.4. Expression of a Ca^2+^-Insensitive Calmodulin Mutant Reduces Gene Transcription Induced by Stimulation of TRPM8 Channels with the Super-Cooling Compound Icilin

**Figure 3:**
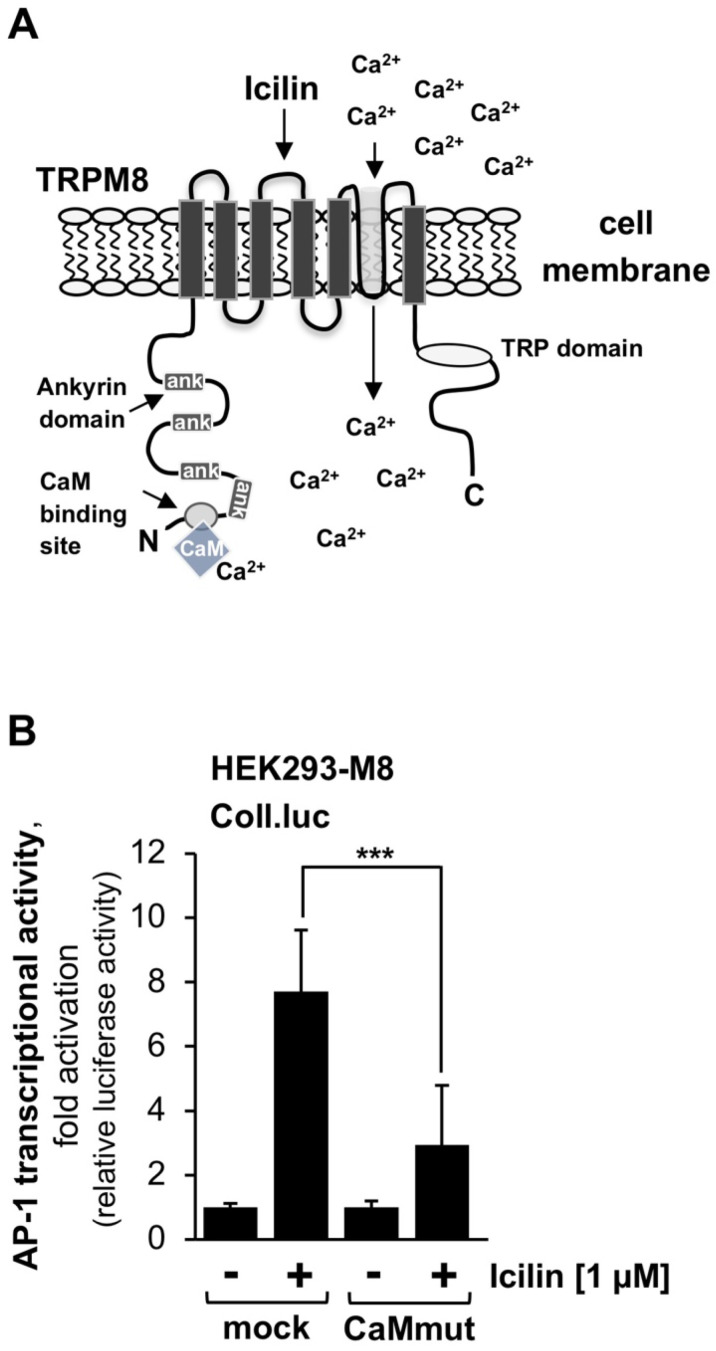
Expression of a calmodulin mutant that is defective in Ca^2+^ binding reduces TRPM8-induced gene transcription. (**A**) Modular structure of TRPM8 showing a proposed calmodulin binding site within the *N*-terminal cytoplasmic domain. (**B**) HEK293–M8 cells containing the Coll.luc reporter gene were infected with a lentivirus encoding either a calmodulin mutant (CaMmut) deficient in Ca^2+^ binding or β-galactosidase (mock) as a control. Cells were serum-starved for 24 h and then stimulated with icilin (1 μM) for 24 h. Cells were harvested and analyzed as described in the legend to Figure 1. Data shown are means ± SD of three experiments performed in quadruplicate (*** *p* < 0.001).

Calmodulin binds to the N-terminal cytoplasmic domain of the TRPM8 channel in vitro, as indicated in the cartoon depicted in Figure 3A. However, experiments involving different calmodulin inhibitors revealed inconsistent results [20,28,29]. We, therefore, tested whether the expression of a calmodulin mutant defective in Ca^2+^ binding alters the signaling cascade of TRPM8 after stimulation with icilin. HEK293-TRPM8 cells, HEK293 cells constitutively expressing TRPM8 channels, were infected with a lentivirus to integrate the reporter gene Coll.luc into the genome of these cells. In addition, cells were infected with a lentivirus encoding either the calmodulin mutant (CaMmut) or β-galactosidase (mock). Figure 3B shows that TRPM8 signaling was significantly impaired in cells expressing the Ca^2+^-insensitive calmodulin mutant. AP-1 activity was reduced by 62.3%. Thus, calmodulin is necessary for the signaling cascade induced by icilin-stimulated TRPM8 channels. When we expressed this mutant specifically in the nucleus, no effect was observed.

### 2.5. TRPM3 and TRPM8 Signaling Is Impaired in the Presence of the Calmodulin Inhibitor Ophiobolin A

**Figure 4:**
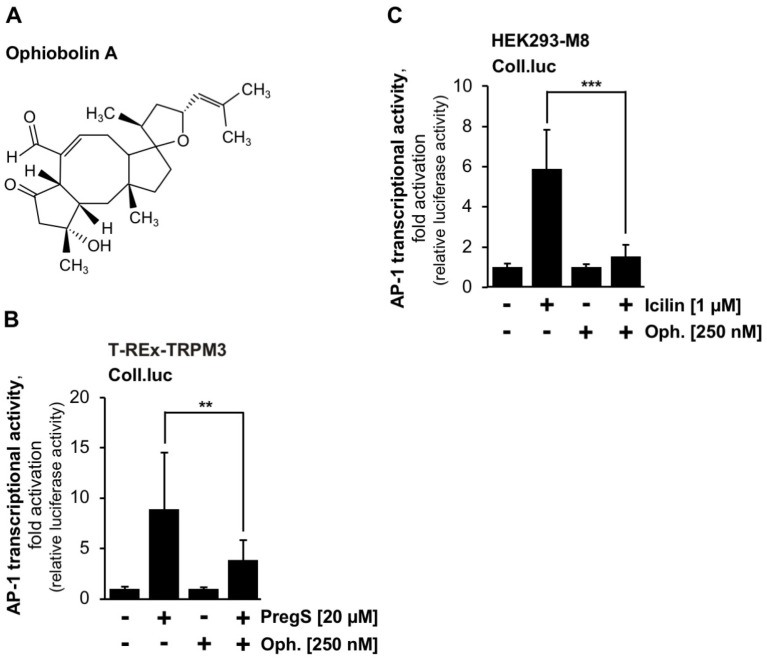
The calmodulin inhibitor ophiobolin A attenuates gene transcription induced by stimulation of TRPM3 and TRPM8 channels. (**A**) Ophiobolin A. (**B**,**C**) T-REx-TRPM3 cells (**B**) and HEK293–M8 cells (**C**), which had the Coll.luc reporter gene integrated into their chromatin, were incubated with ophiobolin A (250 nM) for 3 h in a serum-reduced medium. Cells were stimulated with pregnenolone sulfate (20 μM) (**B**) or icilin (1 μM) (**C**) in the presence or absence of ophiobilin A. Cells were harvested and analyzed as described in the legend to Figure 1 (*n* = 3 (**B**); *n* = 4 (**C**); *** *p* < 0.001; ** *p* < 0.01).

Based on the use of the calmodulin inhibitor ophiobolin A, it has been suggested that calmodulin is required for TRPM8 desensitization [20]. Therefore, we wanted to know whether this compound has an effect on signaling pathways induced by stimulation of TRPM3 or TRPM8 channels. The chemical structure of ophiobolin A is depicted in Figure 4A. T-REx-TRPM3 and HEK293-M8 cells harbouring the Coll.luc reporter gene were preincubated for three hours with the compound and then stimulated with either pregnenolone sulfate or icilin in the presence of ophiobolin A. Titration experiments showed that micromolar concentrations of ophiobolin A are highly toxic, resulting in a complete detachment of the cells. We, therefore, used a concentration of 250 nM for the experiments. Figure 4B,C shows that ophiobolin A significantly reduced gene transcription induced by the stimulation of TRPM3 channels (Figure 4B) or TRPM8 channels (Figure 4C). AP-1 activity was reduced by 56.2% (TRPM3) or 74.6% (TRPM8), respectively. These results indicate that inhibition of calmodulin impairs the signaling cascades of TRPM3 and TRPM8 channels, suggesting that calmodulin is required for linking TRP channel stimulation with gene transcription.

### 2.6. Calcineurin Negatively Regulates TRPM8-Induced Gene Transcription

**Figure 5:**
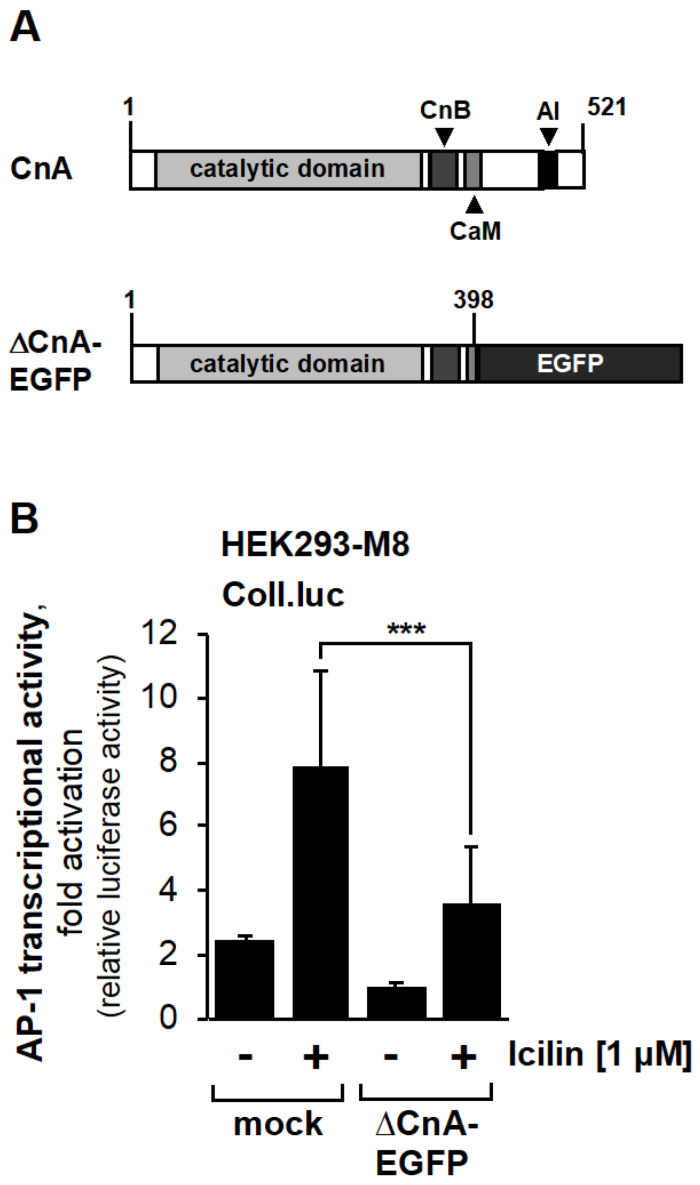
Calcineurin negatively regulates TRPM8-induced signal transduction and gene transcription. (**A**) Domain structure of calcineurin A and CnA–EGFP, a constitutively active mutant of calcineurin A, expressed as a fusion protein EGFP. The binding sites of calmodulin (CaM) and calcineurin B (CnB) are depicted. AI, autoinhibitory domain. (**B**) HEK293–M8 cells containing an integrated Coll-luc reporter gene were infected with a lentivirus encoding either the constitutively active phosphatase (CnA–EGFP) or β-galactosidase (mock) as a control. Cells were serum-starved for 24 h and then stimulated with icilin (1 μM) for 24 h. Cells were harvested and analyzed as described in the legend to Figure 1 (*n* = 5; *** *p* < 0.001).

Calmodulin, as a Ca^2+^/calmodulin complex, regulates the activity of the Ca^2+^/calmodulin-dependent protein phosphatase calcineurin. The domain structure of calcineurin is shown in Figure 5A. The phosphatase contains an autoinhibitory domain (AI) at its C-terminus that binds to the active site of the enzyme in the absence of Ca^2+^/calmodulin. An increase in intracellular Ca^2+^ triggers activation of the enzyme via binding of a Ca^2+^/calmodulin complex to the regulatory region of calcineurin, and by this means displacing the autoinhibitory domain from the active site [39]. Deletion of the autoinhibitory domain and part of the Ca^2+^/calmodulin binding domain leads to the generation of a constitutively active calcineurin mutant that does not require Ca^2+^ or calmodulin for activation. This mutant is depicted in Figure 5A, expressed as a fusion protein with EGFP. A few years ago we showed that expression of this mutant reduces TRPM3 signaling [38]. We, therefore, asked whether calcineurin also has a negative effect on TRPM8 signaling. Figure 5B shows that expression of the constitutively active calcineurin mutant ΔCnA-EGFP significantly reduced icilin-stimulated TRPM8 signaling. AP-1 activity was reduced by 54.4%. Together with previously published data on TRPM3 signaling, we conclude that calcineurin functions as a Ca^2+^ and calmodulin-induced feedback loop to inhibit TRPM3 and TRPM8 signaling and the regulation of gene transcription through these channels.

## 3. Discussion

Many studies have reported that calmodulin binds to various TRP channels and regulates their activities. The aim of this study was to investigate the functional consequences of calmodulin on the signaling cascade after stimulation of TRPM3 or TRPM8 channels. In vitro binding of calmodulin to TRPM3 has been shown [21,22]. Moreover, it has been proposed that mutation of a particular calmodulin binding site reduces TRPM3 activity [22]. However, expression of this mutant was strongly reduced and so was the increase in cytosolic Ca^2+^ concentration after stimulation, used as a measure for TRPM3 activity [22]. Thus, the reduced Ca^2+^ signal was most likely a consequence of the reduced expression level of the TRPM3 mutant. Moreover, no surface biotinylation experiments were performed to verify the expression of the mutated TRPM3 channel at the plasma membrane. Therefore, the expression of the TRPM3 mutant at the plasma membrane might be even lower than shown. It is not known whether the lower stability of this TRPM3 mutant is related to the inactivation of a specific calmodulin binding site. For TRPM8, calmodulin has been proposed to be involved in acute desensitization of the channel. These results were obtained by applying the calmodulin inhibitor ophiobolin A [20]. In contrast, the application of the calmodulin inhibitor calmidazolin did not result in any change in the current elicited by icilin [29].

Our goal was to perform functional studies on the role of calmodulin in TRPM3 and TRPM8 signaling. For this purpose, we used two strategies. We expressed a calmodulin mutant in cells that could no longer bind Ca^2+^ ions because of mutations of all Ca^2+^ binding EF sites. This approach has been widely used by other investigators to study the role of calmodulin in ion channel activity [1,2,4,5,6,7,8,9,13,14,18,32,33,34,35]. In addition, we used a pharmacological approach to inhibit calmodulin. The compound ophiobolin A has been used to analyze the involvement of calmodulin in the regulation of TRPM8 channel activity [20]. Our results show that expression of a calmodulin mutant that was unable to bind Ca^2+^ impairs signal transduction and gene transcription after stimulation of TRPM3 and TRPM8 channels, suggesting that calmodulin is a positive regulator of TRPM3 and TRPM8 channels. The calmodulin mutant functions by competing with wild-type calmodulin for binding to target proteins. Although binding of calmodulin to target proteins often requires binding of a Ca^2+^/calmodulin complex, several reports have shown that apocalmodulin, i.e., calmodulin without bound Ca^2+^ ions, can associate with certain target proteins such as the voltage-gated Ca^2+^ channel or the Ca^2+^-activated K^+^ channel in a Ca^2+^-independent manner. In the case of TRPM3 channels, in vitro experiments with calmodulin-sepharose columns suggest that Ca^2+^-bound calmodulin interacts predominantly with the channel. However, in pull-down experiments, residual binding of Ca^2+^-free calmodulin was observed [22]. Accordingly, the calmodulin mutant can bind to the TRPM3 channel and thus competes with the binding of wild-type calmodulin. Based on the experimental evidence presented here, we also propose that the calmodulin mutant binds to the TRPM8 channel and functions as a dominant-negative. Together with several other reports addressing the regulation of ion channels by calmodulin, we suggest that the regulation of ion channels by calmodulin is dynamic and may involve regulation by both Ca^2+^-free and Ca^2+^-bound calmodulin.

Calmodulin is a small protein that can diffuse through the nuclear pore complex into the nucleus. Regulation of the transcription factor CREB by neuronal activity has been shown to involve calmodulin in the nucleus [36,37]. In fact, calmodulin was detected in the nucleus of hippocampal neurons 1 min after a brief burst of activity [36]. Translocation of calmodulin to the nucleus has also been observed in cholecystokinin or acetylcholine-stimulated pancreatic acinar cells [40]. The results presented here show that the calmodulin mutant targets proteins upstream of B-Raf, suggesting that nuclear calmodulin plays no role in this scenario. The different requirements for nuclear calmodulin in the activation of CREB and AP-1 may be due to the mode of activation of these transcription factors. CREB is phosphorylated by nuclear Ca^2+^/calmodulin-dependent protein kinase IV, which requires nuclear calmodulin for its activation.

We complemented the genetic approach with a pharmacological strategy to inhibit calmodulin. Culturing of spinal ganglion neurons with the calmodulin inhibitor ophiobolin A showed that acute desensitization of TRPM8 channels was significantly attenuated [20]. The authors conclude from these data that calmodulin is a negative regulator of TRPM8 function by forcing the channel protein into a state of low activity leading to acute inactivation. Ophiobolin A concentrations of 100 μM were used in this study. We performed titration experiments that showed that even a concentration of 1 μM was toxic for HEK293 cells expressing TRPM8 or TRPM3 channels. Thus, results obtained with toxic ophiobolin A concentrations of 100 μM are more than questionable. Finally, we used a concentration of 250 nM ophiobolin A for our experiments, which show that application of the calmodulin inhibitor significantly reduced TRPM3 and TRPM8 signaling and gene transcription. These data thus confirm the results obtained by overexpressing a calmodulin mutant incapable of Ca^2+^ binding and show that calmodulin positively regulates TRPM3 and TRPM8 signal transduction and gene transcription.

Calmodulin not only regulates TRP channel activity by direct interaction but can also influence TRP channel signaling by activating calmodulin-dependent enzymes. An important enzyme in the intracellular signaling scheme is the Ca^2+^/calmodulin-dependent protein phosphatase calcineurin. A Ca^2+^/calmodulin complex binds to a well-characterized binding site within the calcineurin A molecule and displaces the C-terminal autoinhibitory domain from the active site, allowing substrate access to the enzyme [39,41]. Using genetically encoded fluorescent biosensors, it has been shown that spatial and temporal calcineurin signaling is dependent on calmodulin [42]. Thus, Ca^2+^ influx via TRPM3 or TRPM8 can lead to the formation of a Ca^2+^/calmodulin complex that subsequently activates calcineurin. While calmodulin in its Ca^2+^-free form can bind to IQ motifs of calmodulin target proteins, proteins with autoinhibitory domains, such as calcineurin and Ca^2+^/calmodulin-dependent protein kinases, are activated under conditions where the intracellular Ca^2+^ concentration is high. The general activation mechanism of these enzymes is based on a Ca^2+^/calmodulin-induced transition from an inactive to an activated form. The calmodulin mutant used in this study mimics apocalmodulin, which should not bind to the autoinhibitory domain protein calcineurin, because of the interaction of the autoinhibitory domain with the catalytic center of the enzyme.

**Figure 6:**
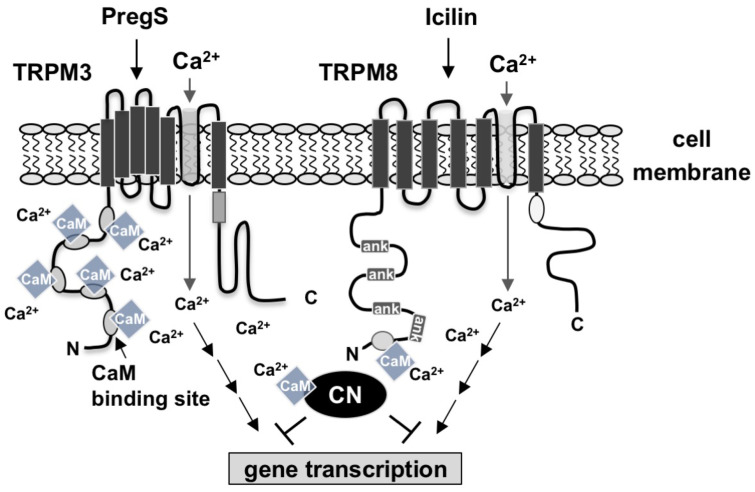
Positive and negative regulation of TRPM3 and TRPM8-induced signal transduction by calmodulin. Calmodulin binds to calmodulin-binding sites of TRPM3 and TRPM8 channels and enhances signal transduction and gene transcription after stimulation of the channels with pregnenolone sulfate or icilin. A Ca^2+^/calmodulin complex activates the phosphatase calcineurin, which negatively regulates TRPM3 and TRPM8 signaling and thus acts as a negative feedback loop.

We have recently shown that signaling via TRPM3 channels is impaired in the presence of a constitutively active calcineurin A mutant. In this study, we showed that calcineurin negatively regulates intracellular signaling via TRPM8 as well. Given that activated calcineurin impairs TRPM3 and TRPM8 signaling and gene transcription, we conclude that calcineurin functions as a negative regulator of TRPM3 and TRPM8 signaling. Experiments involving a constitutively active calcineurin A mutant revealed that calcineurin interferes with the signaling cascade leading to the activation of AP-1 downstream of B-Raf [43]. AP-1 is a homo- or heterodimer of basic region leucine zipper transcription factors, most prominent the c-Fos and c-Jun proteins. Calcineurin dephosphorylates the transcription factor Elk-1, a major regulator of c-Fos gene transcription. Expression of a constitutively active mutant of calcineurin A has been shown to impair c-Fos expression after depolarization of neurons or activation of G protein-coupled receptors [44,45], suggesting that calcineurin interferes with the TRPM3 and TRPM8-induced signaling cascade by preventing c-Fos biosynthesis. Taken together, these data suggest that calmodulin, which is required for calcineurin activation, also functions as a negative regulator of TRPM3 and TRPM8-induced gene transcription via activation of calcineurin (Figure 6).

## 4. Materials and Methods

### 4.1. Cell Culture and Reagents

T-REx-TRPM3 cells and HEK293-M8 cells, HEK293 cells expressing either TRPM3 or TRPM8, respectively, have been described elsewhere [31,46]. HEK293 cells expressing a conditionally active B-Raf kinase mutant (HEK293-ΔB-Raf:ER cells) have been described [47]. The mutant kinase is activatable with 4-hydroxytamoxifen (4OHT). T-REx-TRPM3 cells, HEK293-M8 cells, and HEK293-ΔBRaf:ER were maintained in DMEM supplemented with 0.05% fetal calf serum for 24 h prior to stimulation. Stimulation was performed with pregnenolone sulfate (PregS, 20 μM, dissolved in DMSO, Sigma-Aldrich GmbH, Taufkirchen, Germany, # P162), icilin (1 μM, Santa Cruz Biotechnology, Heidelberg, Germany, # sc-201557), or 4-hydroxytamoxifen (4OHT) (100 nM, Sigma # H7904, with ethanol as solvent, Taufkirchen, Germany) for 24 h in medium supplemented with 0.05% fetal calf serum. Cells were preincubated for 3 h with the compound ophiobolin A (1′*R*,2*S*,3*S*,3′*S*,4′*R*,5*R*,7′*S*,8′*E*,11′*R*)-4′-hydroxy-1′,3,4′-trimethyl-5-(2-methylprop-1-enyl)-6′-oxospiro[oxolane-2,12′-tricyclo[9.3.0.0^3,7^]tetradec-8-ene]-8′-carbaldehyde, PubChem CID 5281387) (Santa Cruz # sc-2022266*,* dissolved in DMSO, Heidelberg, Germany) at a concentration of 250 nM. Cells were stimulated for 24 h in the presence of ophiobolin A.

### 4.2. Lentiviral Gene Transfer

Plasmids pCaMwt-IRES-EGFP and pCaMDN-IRES-EGFP, encoding either wild-type calmodulin or mutant dominant-negative calmodulin, were kindly provided by Marc Shapiro, UT Health Science Center, San Antonio, TX [4,5]. The calmodulin mutant contains alanine substitutions within all four EF-hands (D20A, D56A, D93A, D129A). It has been experimentally confirmed that the mutant cannot bind Ca^2+^ ions [4]. Plasmids pCaMwt-IRES-EGFP and pCaMΔN-IRES-EGFP were cut with SalI, filled in with the Klenow fragment of DNA polymerase I, and cut again with BamHI. The fragments were cloned into the plasmid 3xFLAG-CMV or 3xFLAG-NLS-CMV that had been cut with EcoR1, filled in with the Klenow fragment, and cut again with BamHI, resulting in the plasmids pCMV-FLAG-CaMwt, pCMV-FLAG-CaMmut, pCMV-FLAG-NLS-CaMwt, and pCMV-NLS-FLAG-CaMmut. The coding regions of these plasmids, including the FLAG epitope, were cloned into a lentiviral vector. Lentiviral expression of wild-type and mutant calmodulin had been checked by Western blotting experiments using the FLAG epitope of the recombinant proteins. The lentiviral transfer vector pFUW-ΔCnA-EGFP, encoding a truncated mutant of calcineurin A, has been described elsewhere [38]. Viral particles were prepared as described [48].

### 4.3. Reporter Gene Assay

The lentiviral transfer vector pFWColl.luc has been described [30]. Infected cells were cultured in a medium containing 0.05% fetal calf serum for 24 h prior to stimulation. Reporter lysis buffer (Promega, Mannheim, Germany) was used to prepare cell extracts that were used to determine luciferase activities. These activities were normalized to protein concentrations. We used a luminometer (Berthold Detection Systems, Huntsville, Alabama, USA) to measure the luciferase activities of the extracts. Protein concentrations of the extracts were measured using a BCA protein assay kit.

### 4.4. Statistics

Two-tailed Student’s *t*-test was used for the statistical analyses. The statistical probability is expressed as *** *p <* 0.001; ** *p <* 0.01, and * *p <* 0.05. We considered the values significant when *p <* 0.05.

## 5. Conclusions

Calmodulin is a small Ca^2+^ binding protein that regulates numerous enzymes and ion channels. Using genetic and pharmacological methods, we demonstrated that signal transduction and gene transcription triggered by stimulation of the transient receptor potential channels TRPM3 and TRPM8 are positively regulated by calmodulin, most likely through direct interactions with calmodulin binding sites within the channel proteins. In contrast, no effect of nuclear calmodulin was observed. Calmodulin is also required to activate the Ca^2+^/calmodulin-dependent protein phosphatase calcineurin, which negatively regulates TRPM3 and TRPM8-induced signal transduction and gene transcription. Thus, calmodulin plays a dual role in regulating the functions of TRPM3 and TRPM8 channels: it supports activation of TRPM3 and TRPM8-induced intracellular signaling, most likely through a direct interaction with the channels. Ca^2+^ influx through TRPM3 and TRPM8 feeds back to the TRPM3 and TRPM8 signaling by activation of calcineurin, which acts as a negative feedback loop for both TRPM3 and TRPM8 channel signal transduction and gene transcription.

## Data Availability

Not applicable.
